# (*E*)-2-(2-Chloro-3,3,3-trifluoro­prop-1-en­yl)-*N*-(2,4-dimethyl­phen­yl)-3,3-dimethyl­cyclo­propane-1-carboxamide

**DOI:** 10.1107/S1600536809019813

**Published:** 2009-06-06

**Authors:** Han Zheng, Dong-Qing Liu, Fan-Yong Yan

**Affiliations:** a College of Materials and Chemical Engineering, Tianjin Polytechnic University, Tianjin 300160, People’s Republic of China; b College of Materials and Chemical Engineering, Tianjin Key Laboratory of Fiber Modification & Functional Fibers, Tianjin Polytechnic University, Tianjin 300160, People’s Republic of China

## Abstract

The title compound, C_17_H_19_ClF_3_NO, crystallizes with three mol­ecules in the asymmetric unit. The aromatic ring makes dihedral angles of 38.69 (13), 46.68 (12) and 50.52 (11)° with the plane of the cyclo­propane ring in the three mol­ecules. The crystal packing is stabilized by inter­molecular N—H⋯O hydrogen bonds.

## Related literature

For the synthesis, see: Liu *et al.* (2006 ▶). The title compound is an inter­mediate for the insecticide tefluthrin, see: Punja (1981 ▶). Pesticides containing 2,4-dimethyl­benzenamine have the advantage of low toxicity, high activity and low residues (Zhang 2005 ▶).
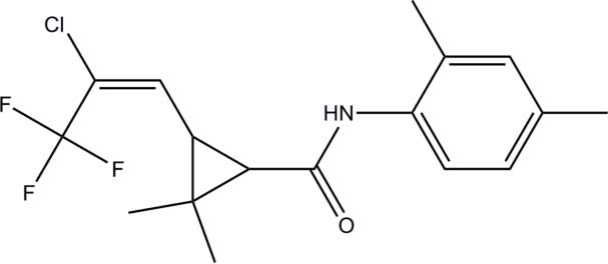


## Experimental

### 

#### Crystal data

C_17_H_19_ClF_3_NO
*M* *_r_* = 345.78
Triclinic, 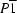

*a* = 8.8497 (18) Å
*b* = 12.686 (3) Å
*c* = 23.891 (5) Å
α = 98.11 (3)°
β = 94.51 (3)°
γ = 97.60 (3)°
*V* = 2619.1 (9) Å^3^
*Z* = 6
Mo *K*α radiation
μ = 0.25 mm^−1^
*T* = 113 K
0.40 × 0.12 × 0.10 mm

#### Data collection

Rigaku Saturn CCD area-detector diffractometer
Absorption correction: multi-scan (*CrystalClear*; Rigaku/MSC, 2005 ▶) *T*_min_ = 0.907, *T*_max_ = 0.975
19478 measured reflections
9213 independent reflections
6737 reflections with *I* > 2σ(*I*)
*R*_int_ = 0.036

#### Refinement

*R*[*F* ^2^ > 2σ(*F* ^2^)] = 0.050
*wR*(*F* ^2^) = 0.137
*S* = 1.01
9213 reflections
684 parameters
81 restraints
H atoms treated by a mixture of independent and constrained refinement
Δρ_max_ = 0.35 e Å^−3^
Δρ_min_ = −0.39 e Å^−3^

### 

Data collection: *CrystalClear* (Rigaku/MSC, 2005 ▶); cell refinement: *CrystalClear*; data reduction: *CrystalClear*; program(s) used to solve structure: *SHELXS97* (Sheldrick, 2008 ▶); program(s) used to refine structure: *SHELXL97* (Sheldrick, 2008 ▶); molecular graphics: *SHELXTL* (Sheldrick, 2008 ▶); software used to prepare material for publication: *SHELXTL*.

## Supplementary Material

Crystal structure: contains datablocks I, global. DOI: 10.1107/S1600536809019813/bt2966sup1.cif

Structure factors: contains datablocks I. DOI: 10.1107/S1600536809019813/bt2966Isup2.hkl

Additional supplementary materials:  crystallographic information; 3D view; checkCIF report

## Figures and Tables

**Table 1:** Hydrogen-bond geometry (Å, °)

*D*—H⋯*A*	*D*—H	H⋯*A*	*D*⋯*A*	*D*—H⋯*A*
N1—H1*A*⋯O2	0.897 (10)	1.950 (11)	2.840 (2)	171 (2)
N2—H2*A*⋯O3	0.91	1.99	2.893 (2)	174
N3—H3*A*⋯O1^i^	0.90	2.10	2.980 (2)	167

Symmetry code: (i) 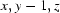.

**Fig. 1.**
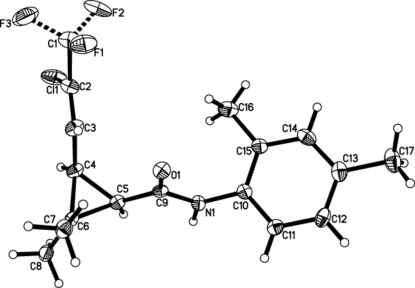
The molecular structure of one molecule in the asymmetric unit, drawn with 30% probability ellipsoids. H atoms are drawn as spheres of arbitrary radius.

## supplementary crystallographic information

### Comment

3-((*E*)-2-chloro-3,3,3-trifluoroprop-1-enyl)-2,2-dimethyl cyclopropanecarboxylic acid is a very important intermediate for tefluthrin, a important insecticide controlling a wide range of soil insect pests in maize,sugar beet, and other crops (Punja 1981). 2,4-dimethylbenzenamine containing pesticides have the advantage of low toxicity, high activity and low residues (Zhang 2005). The structure in this article containing both of two active parts may be show some insecticide activity probably. The present X-ray crystal structure analysis was undertaken in order to study the stereochemistry and crystal packing of the title compound, (I) In this paper, the title compound, (*E*)-3-(2-chloro-3,3,3-trifluoroprop-1-enyl)-*N*-(2,4-dimethylphenyl)- 2,2-dimethylcyclopropanecarboxamide, (I), was synthesized and the structure of (I) was illustrated in Fig. 1. The dihedral angles between the benzene moiety and the cycloprapane groug is 38.6 (6)°. The amide hydrogen was linking with the amide oxygen in another molecule by an intermolecular N—H···O hydrogen bond. The packing can be described as shown in Fig. 2 and Table 1.

### Experimental

The title compound (I) was prepared according to the method of Liu *et al.* (2006). 3-((*E*)-2-chloro-3,3,3-trifluoroprop-1-enyl)-2,2- dimethylcyclopropanecarboxylic acid (0.97 g, 4.0 mmol) was dispersed in SOCl_2_ (19 ml), and a drop of anhydrous DMF was added in. The mixture was heated to reflux for 3 h. SOCl_2_ was removed by rotoevapor. The crude the product could be directly disolved in anhydrous toluene, mixed with 2,4-dimethylbenzenamine (0.50 g, 4.1 mmol) already. Triethylamine was dropped into the system, After 12 h stirring under room temperature, the reaction mixture was treated with hexane. The product was recrystallized from methanol and ethyl acetate (15:1) over 4 d at ambient temperature, gave rod colourless crystals of (*E*)-3-(2-chloro-3,3,3-trifluoroprop-1-enyl)-N– (2,4-dimethylphenyl)- 2,2-dimethylcyclopropanecarboxamide, suitable for X-ray analysis.

### Refinement

H atoms were positioned geometrically with C—H = 0.93–0.98 Å and refined using riding model with *U*_iso_(H) = 1.2Ueq(C). The H atom of one N—H was located from difference map and refined freely. The other two were refined using riding model with N-H = 0.91Å and *U*_iso_(H) = 1.2Ueq(N). One CF_3_ group and one Cl ligand are disordered over two positions with site occupation factors of 0.61 (3)/0.39 (3) and 0.676 (8)/0.324 (8), respectively. Restraints were applied to keep the geometric parameters in a reasonable range.

### Crystal data

**Table:**

C_17_H_19_ClF_3_NO	*F*(000) = 1080
*M**_r_* = 345.78	*D*_x_ = 1.315 Mg m^−^^3^
Triclinic, *P*1	Melting point = 366–368 K
*a* = 8.8497 (18) Å	Mo *K*α radiation, λ = 0.71073 Å
*b* = 12.686 (3) Å	Cell parameters from 6424 reflections
*c* = 23.891 (5) Å	θ = 1.7–27.5°
α = 98.11 (3)°	µ = 0.25 mm^−^^1^
β = 94.51 (3)°	*T* = 113 K
γ = 97.60 (3)°	Rod, colorless
*V* = 2619.1 (9) Å^3^	0.40 × 0.12 × 0.10 mm
*Z* = 6	

### Data collection

**Table:**

Rigaku Saturn CCD area-detector diffractometer	9213 independent reflections
Radiation source: rotating anode	6737 reflections with *I* > 2σ(*I*)
confocal	*R*_int_ = 0.036
Detector resolution: 7.31 pixels mm^-1^	θ_max_ = 25.0°, θ_min_ = 1.7°
ω and φ scans	*h* = −8→10
Absorption correction: multi-scan (*CrystalClear*; Rigaku/MSC, 2005)	*k* = −14→15
*T*_min_ = 0.907, *T*_max_ = 0.975	*l* = −26→28
19478 measured reflections	

### Refinement

**Table:**

Refinement on *F*^2^	Primary atom site location: structure-invariant direct methods
Least-squares matrix: full	Secondary atom site location: difference Fourier map
*R*[*F*^2^ > 2σ(*F*^2^)] = 0.050	Hydrogen site location: inferred from neighbouring sites
*wR*(*F*^2^) = 0.137	H atoms treated by a mixture of independent and constrained refinement
*S* = 1.01	*w* = 1/[σ^2^(*F*_o_^2^) + (0.0784*P*)^2^] where *P* = (*F*_o_^2^ + 2*F*_c_^2^)/3
9213 reflections	(Δ/σ)_max_ = 0.001
684 parameters	Δρ_max_ = 0.35 e Å^−^^3^
81 restraints	Δρ_min_ = −0.39 e Å^−^^3^

### Special details

**Table:**

Geometry. All e.s.d.'s (except the e.s.d. in the dihedral angle between two l.s. planes) are estimated using the full covariance matrix. The cell e.s.d.'s are taken into account individually in the estimation of e.s.d.'s in distances, angles and torsion angles; correlations between e.s.d.'s in cell parameters are only used when they are defined by crystal symmetry. An approximate (isotropic) treatment of cell e.s.d.'s is used for estimating e.s.d.'s involving l.s. planes.
Refinement. Refinement of *F*^2^ against ALL reflections. The weighted *R*-factor *wR* and goodness of fit *S* are based on *F*^2^, conventional *R*-factors *R* are based on *F*, with *F* set to zero for negative *F*^2^. The threshold expression of *F*^2^ > σ(*F*^2^) is used only for calculating *R*-factors(gt) *etc*. and is not relevant to the choice of reflections for refinement. *R*-factors based on *F*^2^ are statistically about twice as large as those based on *F*, and *R*- factors based on ALL data will be even larger.

### Fractional atomic coordinates and isotropic or equivalent isotropic displacement parameters (Å^2^)

**Table:**

	*x*	*y*	*z*	*U*_iso_*/*U*_eq_	Occ. (<1)
Cl1	0.1893 (5)	0.70100 (11)	0.05532 (6)	0.0636 (9)	0.676 (8)
Cl1'	0.2962 (12)	0.7294 (6)	0.06524 (18)	0.080 (2)	0.324 (8)
Cl2	1.16008 (7)	0.77856 (6)	0.47489 (3)	0.0481 (2)	
Cl3	0.09570 (7)	0.27758 (5)	0.42419 (2)	0.03712 (17)	
F1	0.1708 (15)	1.0056 (5)	0.0636 (4)	0.0613 (19)	0.61 (3)
F2	0.2499 (13)	0.8969 (8)	−0.0009 (4)	0.073 (2)	0.61 (3)
F3	0.0176 (9)	0.8747 (9)	0.0148 (4)	0.079 (2)	0.61 (3)
F1'	0.112 (2)	0.9913 (11)	0.0630 (6)	0.062 (3)	0.39 (3)
F2'	0.2885 (14)	0.9340 (17)	0.0156 (8)	0.081 (3)	0.39 (3)
F3'	0.059 (2)	0.8424 (12)	0.0040 (5)	0.089 (4)	0.39 (3)
F4	1.27615 (18)	0.95865 (14)	0.41326 (7)	0.0614 (5)	
F5	1.1713 (2)	1.02023 (13)	0.48606 (7)	0.0680 (5)	
F6	1.0625 (2)	1.01829 (12)	0.40246 (8)	0.0671 (5)	
F7	0.00296 (17)	0.46689 (13)	0.37332 (8)	0.0616 (5)	
F8	0.1468 (2)	0.52082 (13)	0.45145 (7)	0.0659 (5)	
F9	0.23130 (17)	0.54775 (11)	0.37203 (7)	0.0475 (4)	
O1	0.46317 (18)	1.02240 (12)	0.20361 (6)	0.0324 (4)	
O2	0.73814 (19)	0.78140 (12)	0.30027 (6)	0.0336 (4)	
O3	0.58314 (18)	0.39807 (12)	0.29590 (6)	0.0289 (4)	
N1	0.6568 (2)	0.93710 (15)	0.23332 (8)	0.0295 (4)	
N2	0.6703 (2)	0.60563 (14)	0.26159 (7)	0.0246 (4)	
N3	0.6016 (2)	0.25305 (15)	0.23034 (7)	0.0268 (4)	
C1	0.1621 (4)	0.9022 (2)	0.04166 (11)	0.0541 (8)	
C2	0.2011 (3)	0.8357 (2)	0.08589 (10)	0.0394 (6)	
C3	0.2182 (3)	0.8697 (2)	0.14038 (10)	0.0390 (6)	
H3	0.2040	0.9404	0.1525	0.047*	
C4	0.2583 (3)	0.80473 (19)	0.18437 (9)	0.0330 (6)	
H4	0.2357	0.7267	0.1724	0.040*	
C5	0.4048 (3)	0.84262 (18)	0.22563 (9)	0.0288 (5)	
H5	0.4587	0.7841	0.2356	0.035*	
C6	0.2473 (3)	0.84135 (18)	0.24663 (9)	0.0302 (5)	
C7	0.1860 (3)	0.9448 (2)	0.26512 (11)	0.0389 (6)	
H7A	0.2203	0.9971	0.2418	0.058*	
H7B	0.2225	0.9714	0.3041	0.058*	
H7C	0.0760	0.9316	0.2612	0.058*	
C8	0.2028 (3)	0.7533 (2)	0.28160 (10)	0.0381 (6)	
H8A	0.2497	0.7747	0.3198	0.057*	
H8B	0.2371	0.6881	0.2653	0.057*	
H8C	0.0935	0.7414	0.2818	0.057*	
C9	0.5075 (3)	0.94219 (18)	0.21988 (9)	0.0267 (5)	
C10	0.7772 (3)	1.01967 (18)	0.22573 (9)	0.0283 (5)	
C11	0.8607 (3)	1.08240 (19)	0.27332 (10)	0.0330 (6)	
H11	0.8339	1.0734	0.3094	0.040*	
C12	0.9832 (3)	1.1580 (2)	0.26734 (11)	0.0385 (6)	
H12	1.0382	1.1998	0.2995	0.046*	
C13	1.0253 (3)	1.1722 (2)	0.21380 (12)	0.0391 (6)	
C14	0.9381 (3)	1.1105 (2)	0.16684 (11)	0.0399 (6)	
H14	0.9639	1.1211	0.1308	0.048*	
C15	0.8137 (3)	1.03322 (19)	0.17109 (10)	0.0323 (6)	
C16	0.7241 (3)	0.9688 (2)	0.11924 (10)	0.0481 (7)	
H16A	0.7026	0.8949	0.1247	0.072*	
H16B	0.7824	0.9735	0.0872	0.072*	
H16C	0.6296	0.9964	0.1124	0.072*	
C17	1.1642 (3)	1.2511 (2)	0.20695 (14)	0.0569 (8)	
H17A	1.2523	1.2145	0.2049	0.085*	
H17B	1.1817	1.3079	0.2389	0.085*	
H17C	1.1468	1.2811	0.1727	0.085*	
C18	1.1401 (3)	0.9619 (2)	0.43435 (11)	0.0445 (7)	
C19	1.0569 (3)	0.85327 (18)	0.43524 (9)	0.0280 (5)	
C20	0.9184 (3)	0.81789 (18)	0.40944 (9)	0.0269 (5)	
H20	0.8743	0.8627	0.3874	0.032*	
C21	0.8287 (2)	0.71443 (17)	0.41270 (8)	0.0244 (5)	
H21	0.8793	0.6708	0.4370	0.029*	
C22	0.7221 (2)	0.64828 (17)	0.36250 (9)	0.0259 (5)	
H22	0.7171	0.5702	0.3606	0.031*	
C23	0.6562 (3)	0.70290 (19)	0.41385 (9)	0.0303 (5)	
C24	0.5926 (3)	0.6299 (2)	0.45425 (10)	0.0448 (7)	
H24A	0.5974	0.6705	0.4916	0.067*	
H24B	0.6521	0.5721	0.4555	0.067*	
H24C	0.4880	0.6008	0.4412	0.067*	
C25	0.5733 (3)	0.7977 (2)	0.40975 (11)	0.0436 (7)	
H25A	0.6130	0.8366	0.3811	0.065*	
H25B	0.5878	0.8441	0.4457	0.065*	
H25C	0.4659	0.7730	0.3998	0.065*	
C26	0.7118 (2)	0.68547 (17)	0.30611 (9)	0.0252 (5)	
C27	0.6653 (3)	0.62123 (17)	0.20348 (8)	0.0257 (5)	
C28	0.7859 (3)	0.68320 (19)	0.18391 (10)	0.0328 (6)	
H28	0.8687	0.7173	0.2093	0.039*	
C29	0.7843 (3)	0.6947 (2)	0.12749 (10)	0.0420 (7)	
H29	0.8658	0.7366	0.1152	0.050*	
C30	0.6631 (4)	0.6448 (2)	0.08916 (10)	0.0480 (7)	
C31	0.5446 (3)	0.5821 (2)	0.10882 (10)	0.0421 (7)	
H31	0.4635	0.5471	0.0829	0.050*	
C32	0.5411 (3)	0.56908 (18)	0.16563 (9)	0.0292 (5)	
C33	0.4088 (3)	0.5011 (2)	0.18502 (10)	0.0374 (6)	
H33A	0.3261	0.4846	0.1552	0.056*	
H33B	0.3752	0.5398	0.2181	0.056*	
H33C	0.4407	0.4355	0.1941	0.056*	
C34	0.6602 (5)	0.6578 (3)	0.02673 (11)	0.0816 (12)	
H34A	0.6963	0.7313	0.0236	0.122*	
H34B	0.5573	0.6384	0.0089	0.122*	
H34C	0.7253	0.6118	0.0083	0.122*	
C35	0.1473 (3)	0.4760 (2)	0.39733 (11)	0.0382 (6)	
C36	0.2059 (3)	0.37096 (18)	0.39228 (9)	0.0286 (5)	
C37	0.3284 (2)	0.35039 (18)	0.36670 (9)	0.0281 (5)	
H37	0.3838	0.4066	0.3526	0.034*	
C38	0.3833 (2)	0.24578 (17)	0.35896 (9)	0.0251 (5)	
H38	0.3056	0.1848	0.3620	0.030*	
C39	0.4964 (2)	0.22032 (17)	0.31554 (8)	0.0248 (5)	
H39	0.4778	0.1458	0.2960	0.030*	
C40	0.5456 (2)	0.23111 (17)	0.37855 (9)	0.0253 (5)	
C41	0.6566 (3)	0.32675 (19)	0.40892 (9)	0.0312 (6)	
H41A	0.7597	0.3142	0.4039	0.047*	
H41B	0.6430	0.3366	0.4487	0.047*	
H41C	0.6377	0.3901	0.3934	0.047*	
C42	0.5648 (3)	0.12680 (19)	0.40059 (10)	0.0342 (6)	
H42A	0.4944	0.0690	0.3786	0.051*	
H42B	0.5443	0.1331	0.4397	0.051*	
H42C	0.6678	0.1123	0.3974	0.051*	
C43	0.5625 (2)	0.29966 (18)	0.28017 (9)	0.0243 (5)	
C44	0.6903 (3)	0.30899 (18)	0.19294 (9)	0.0287 (5)	
C45	0.8212 (3)	0.38029 (19)	0.21508 (10)	0.0346 (6)	
H45	0.8484	0.3942	0.2541	0.042*	
C46	0.9112 (3)	0.4306 (2)	0.17886 (11)	0.0428 (7)	
H46	0.9974	0.4794	0.1940	0.051*	
C47	0.8754 (3)	0.4096 (2)	0.12100 (12)	0.0463 (7)	
C48	0.7443 (3)	0.3385 (2)	0.09992 (11)	0.0472 (7)	
H48	0.7186	0.3246	0.0608	0.057*	
C49	0.6486 (3)	0.28675 (19)	0.13466 (10)	0.0356 (6)	
C50	0.5068 (3)	0.2126 (2)	0.10972 (10)	0.0509 (8)	
H50A	0.4967	0.1504	0.1285	0.076*	
H50B	0.5131	0.1907	0.0699	0.076*	
H50C	0.4192	0.2491	0.1146	0.076*	
C51	0.9757 (4)	0.4620 (3)	0.08105 (13)	0.0714 (10)	
H51A	1.0371	0.5260	0.1014	0.107*	
H51B	0.9124	0.4802	0.0504	0.107*	
H51C	1.0414	0.4128	0.0660	0.107*	
H1A	0.676 (3)	0.8827 (14)	0.2518 (9)	0.039 (7)*	
H2A	0.638 (3)	0.5393 (11)	0.2698 (10)	0.041 (7)*	
H3A	0.574 (3)	0.1820 (9)	0.2195 (10)	0.045 (8)*	

### Atomic displacement parameters (Å^2^)

**Table:**

	*U*^11^	*U*^22^	*U*^33^	*U*^12^	*U*^13^	*U*^23^
Cl1	0.117 (2)	0.0428 (7)	0.0307 (6)	0.0123 (9)	0.0146 (9)	−0.0016 (5)
Cl1'	0.133 (6)	0.090 (3)	0.0398 (17)	0.073 (4)	0.031 (2)	0.0199 (19)
Cl2	0.0293 (3)	0.0594 (5)	0.0548 (4)	−0.0032 (3)	−0.0091 (3)	0.0227 (3)
Cl3	0.0333 (3)	0.0380 (4)	0.0410 (3)	0.0003 (3)	0.0121 (3)	0.0091 (3)
F1	0.085 (5)	0.051 (2)	0.054 (2)	0.023 (2)	−0.003 (3)	0.0204 (17)
F2	0.114 (4)	0.075 (4)	0.043 (3)	0.023 (3)	0.035 (3)	0.029 (3)
F3	0.084 (3)	0.096 (5)	0.056 (3)	0.011 (3)	−0.026 (3)	0.024 (3)
F1'	0.079 (7)	0.073 (5)	0.045 (3)	0.048 (4)	0.002 (5)	0.015 (4)
F2'	0.102 (5)	0.098 (7)	0.061 (6)	0.030 (5)	0.028 (4)	0.044 (5)
F3'	0.125 (7)	0.096 (6)	0.040 (4)	0.029 (5)	−0.033 (4)	0.002 (4)
F4	0.0499 (10)	0.0660 (11)	0.0605 (10)	−0.0260 (9)	0.0196 (8)	0.0072 (8)
F5	0.0725 (12)	0.0559 (11)	0.0570 (10)	−0.0266 (10)	0.0097 (9)	−0.0234 (8)
F6	0.0767 (13)	0.0301 (9)	0.0916 (13)	−0.0089 (9)	−0.0045 (11)	0.0217 (9)
F7	0.0323 (9)	0.0486 (10)	0.1070 (14)	0.0136 (8)	0.0011 (9)	0.0182 (9)
F8	0.0949 (15)	0.0419 (10)	0.0622 (11)	0.0163 (10)	0.0288 (10)	−0.0058 (8)
F9	0.0457 (9)	0.0296 (8)	0.0698 (10)	0.0068 (7)	0.0119 (8)	0.0123 (7)
O1	0.0370 (9)	0.0228 (9)	0.0399 (9)	0.0114 (7)	0.0008 (7)	0.0081 (7)
O2	0.0490 (10)	0.0204 (9)	0.0299 (8)	0.0023 (8)	−0.0064 (7)	0.0065 (7)
O3	0.0403 (9)	0.0182 (8)	0.0286 (8)	0.0021 (7)	0.0085 (7)	0.0048 (6)
N1	0.0299 (11)	0.0238 (11)	0.0372 (11)	0.0058 (9)	0.0013 (9)	0.0125 (9)
N2	0.0300 (10)	0.0218 (10)	0.0213 (9)	0.0011 (8)	0.0015 (8)	0.0047 (8)
N3	0.0357 (11)	0.0216 (10)	0.0253 (10)	0.0085 (9)	0.0061 (8)	0.0052 (8)
C1	0.068 (2)	0.059 (2)	0.0385 (16)	0.0153 (18)	0.0077 (16)	0.0139 (15)
C2	0.0487 (16)	0.0383 (15)	0.0350 (14)	0.0110 (13)	0.0112 (12)	0.0103 (11)
C3	0.0480 (16)	0.0288 (14)	0.0379 (14)	0.0046 (12)	−0.0073 (12)	0.0041 (11)
C4	0.0421 (14)	0.0229 (12)	0.0315 (12)	0.0031 (11)	−0.0060 (11)	0.0025 (10)
C5	0.0332 (13)	0.0227 (12)	0.0321 (12)	0.0099 (10)	0.0000 (10)	0.0060 (9)
C6	0.0271 (12)	0.0287 (13)	0.0335 (12)	0.0051 (10)	−0.0038 (10)	0.0032 (10)
C7	0.0331 (14)	0.0405 (15)	0.0434 (14)	0.0113 (12)	0.0019 (11)	0.0027 (12)
C8	0.0328 (14)	0.0400 (15)	0.0398 (14)	−0.0004 (12)	−0.0021 (11)	0.0093 (11)
C9	0.0350 (13)	0.0232 (12)	0.0219 (11)	0.0074 (10)	0.0008 (9)	0.0013 (9)
C10	0.0288 (12)	0.0244 (12)	0.0355 (13)	0.0105 (10)	0.0030 (10)	0.0114 (10)
C11	0.0297 (13)	0.0359 (14)	0.0354 (13)	0.0120 (11)	−0.0007 (10)	0.0071 (11)
C12	0.0290 (13)	0.0337 (14)	0.0503 (16)	0.0054 (11)	−0.0066 (12)	0.0027 (12)
C13	0.0264 (13)	0.0344 (15)	0.0605 (17)	0.0111 (11)	0.0010 (12)	0.0164 (13)
C14	0.0422 (15)	0.0427 (16)	0.0445 (15)	0.0194 (13)	0.0146 (12)	0.0212 (12)
C15	0.0369 (14)	0.0275 (13)	0.0352 (13)	0.0099 (11)	0.0019 (11)	0.0099 (10)
C16	0.066 (2)	0.0431 (17)	0.0362 (14)	0.0103 (15)	0.0080 (13)	0.0065 (12)
C17	0.0334 (15)	0.0533 (19)	0.088 (2)	0.0025 (14)	0.0046 (15)	0.0278 (17)
C18	0.0459 (16)	0.0425 (16)	0.0371 (14)	−0.0138 (14)	0.0049 (12)	−0.0031 (12)
C19	0.0290 (12)	0.0322 (13)	0.0208 (11)	−0.0016 (10)	0.0034 (9)	0.0026 (9)
C20	0.0320 (13)	0.0242 (12)	0.0235 (11)	0.0022 (10)	−0.0011 (9)	0.0039 (9)
C21	0.0273 (12)	0.0238 (12)	0.0208 (11)	0.0002 (10)	−0.0026 (9)	0.0050 (9)
C22	0.0308 (12)	0.0201 (12)	0.0247 (11)	−0.0005 (10)	−0.0036 (9)	0.0037 (9)
C23	0.0257 (12)	0.0338 (14)	0.0281 (12)	−0.0015 (11)	−0.0007 (10)	0.0007 (10)
C24	0.0396 (15)	0.0579 (18)	0.0295 (13)	−0.0167 (14)	0.0032 (11)	0.0038 (12)
C25	0.0327 (14)	0.0542 (18)	0.0411 (14)	0.0111 (13)	0.0020 (11)	−0.0065 (12)
C26	0.0246 (12)	0.0226 (12)	0.0276 (11)	0.0038 (10)	−0.0010 (9)	0.0031 (9)
C27	0.0327 (13)	0.0225 (12)	0.0232 (11)	0.0076 (10)	0.0030 (10)	0.0044 (9)
C28	0.0355 (14)	0.0318 (14)	0.0325 (13)	0.0057 (11)	0.0035 (10)	0.0088 (10)
C29	0.0512 (17)	0.0420 (16)	0.0358 (14)	0.0062 (13)	0.0152 (12)	0.0112 (12)
C30	0.070 (2)	0.0449 (17)	0.0302 (14)	0.0081 (15)	0.0089 (13)	0.0076 (12)
C31	0.0575 (18)	0.0361 (15)	0.0286 (13)	0.0062 (13)	−0.0080 (12)	−0.0015 (11)
C32	0.0352 (13)	0.0243 (12)	0.0282 (12)	0.0085 (10)	−0.0008 (10)	0.0023 (9)
C33	0.0348 (14)	0.0352 (14)	0.0386 (13)	0.0010 (11)	−0.0066 (11)	0.0024 (11)
C34	0.123 (3)	0.088 (3)	0.0298 (16)	−0.003 (2)	0.0082 (18)	0.0152 (16)
C35	0.0344 (14)	0.0333 (14)	0.0475 (15)	0.0055 (12)	0.0086 (12)	0.0053 (12)
C36	0.0260 (12)	0.0263 (13)	0.0322 (12)	0.0000 (10)	0.0022 (10)	0.0033 (10)
C37	0.0261 (12)	0.0290 (13)	0.0271 (11)	−0.0031 (10)	−0.0025 (10)	0.0062 (10)
C38	0.0229 (11)	0.0235 (12)	0.0285 (11)	−0.0005 (10)	0.0018 (9)	0.0065 (9)
C39	0.0294 (12)	0.0189 (11)	0.0259 (11)	0.0017 (9)	0.0016 (9)	0.0052 (9)
C40	0.0228 (11)	0.0257 (12)	0.0281 (11)	0.0005 (10)	0.0026 (9)	0.0090 (9)
C41	0.0284 (12)	0.0351 (14)	0.0287 (12)	−0.0032 (11)	−0.0015 (10)	0.0092 (10)
C42	0.0329 (13)	0.0341 (14)	0.0382 (13)	0.0028 (11)	0.0024 (11)	0.0174 (11)
C43	0.0250 (12)	0.0249 (13)	0.0236 (11)	0.0054 (10)	−0.0008 (9)	0.0058 (9)
C44	0.0374 (13)	0.0251 (12)	0.0294 (12)	0.0152 (11)	0.0111 (10)	0.0091 (10)
C45	0.0371 (14)	0.0328 (14)	0.0385 (13)	0.0144 (11)	0.0099 (11)	0.0090 (11)
C46	0.0383 (15)	0.0393 (16)	0.0565 (17)	0.0129 (12)	0.0189 (13)	0.0130 (13)
C47	0.0493 (17)	0.0477 (17)	0.0544 (17)	0.0228 (14)	0.0296 (14)	0.0221 (14)
C48	0.0652 (19)	0.0542 (18)	0.0323 (14)	0.0277 (16)	0.0201 (13)	0.0147 (13)
C49	0.0490 (16)	0.0320 (14)	0.0305 (13)	0.0189 (12)	0.0106 (11)	0.0051 (10)
C50	0.069 (2)	0.0530 (18)	0.0292 (13)	0.0110 (16)	0.0025 (13)	0.0020 (12)
C51	0.078 (2)	0.081 (3)	0.070 (2)	0.021 (2)	0.0440 (19)	0.0300 (19)

### Geometric parameters (Å, °)

**Table:**

Cl1—C2	1.748 (3)	C21—C23	1.517 (3)
Cl1'—C2	1.717 (4)	C21—C22	1.537 (3)
Cl2—C19	1.725 (2)	C21—H21	0.9800
Cl3—C36	1.732 (2)	C22—C26	1.489 (3)
F1—C1	1.332 (6)	C22—C23	1.518 (3)
F2—C1	1.327 (6)	C22—H22	0.9800
F3—C1	1.364 (6)	C23—C25	1.500 (4)
F1'—C1	1.318 (8)	C23—C24	1.519 (3)
F2'—C1	1.365 (8)	C24—H24A	0.9600
F3'—C1	1.306 (8)	C24—H24B	0.9600
F4—C18	1.344 (3)	C24—H24C	0.9600
F5—C18	1.336 (3)	C25—H25A	0.9600
F6—C18	1.328 (3)	C25—H25B	0.9600
F7—C35	1.342 (3)	C25—H25C	0.9600
F8—C35	1.337 (3)	C27—C28	1.391 (3)
F9—C35	1.339 (3)	C27—C32	1.395 (3)
O1—C9	1.238 (3)	C28—C29	1.376 (3)
O2—C26	1.238 (2)	C28—H28	0.9300
O3—C43	1.237 (3)	C29—C30	1.376 (4)
N1—C9	1.347 (3)	C29—H29	0.9300
N1—C10	1.433 (3)	C30—C31	1.384 (4)
N1—H1A	0.897 (10)	C30—C34	1.522 (4)
N2—C26	1.352 (3)	C31—C32	1.392 (3)
N2—C27	1.428 (3)	C31—H31	0.9300
N2—H2A	0.905 (10)	C32—C33	1.503 (3)
N3—C43	1.344 (3)	C33—H33A	0.9600
N3—C44	1.435 (3)	C33—H33B	0.9600
N3—H3A	0.897 (10)	C33—H33C	0.9600
C1—C2	1.489 (4)	C34—H34A	0.9600
C2—C3	1.303 (3)	C34—H34B	0.9600
C3—C4	1.474 (3)	C34—H34C	0.9600
C3—H3	0.9300	C35—C36	1.486 (3)
C4—C6	1.509 (3)	C36—C37	1.324 (3)
C4—C5	1.544 (3)	C37—C38	1.465 (3)
C4—H4	0.9800	C37—H37	0.9300
C5—C9	1.485 (3)	C38—C40	1.519 (3)
C5—C6	1.517 (3)	C38—C39	1.529 (3)
C5—H5	0.9800	C38—H38	0.9800
C6—C7	1.508 (3)	C39—C43	1.494 (3)
C6—C8	1.516 (3)	C39—C40	1.516 (3)
C7—H7A	0.9600	C39—H39	0.9800
C7—H7B	0.9600	C40—C41	1.512 (3)
C7—H7C	0.9600	C40—C42	1.516 (3)
C8—H8A	0.9600	C41—H41A	0.9600
C8—H8B	0.9600	C41—H41B	0.9600
C8—H8C	0.9600	C41—H41C	0.9600
C10—C11	1.389 (3)	C42—H42A	0.9600
C10—C15	1.397 (3)	C42—H42B	0.9600
C11—C12	1.380 (3)	C42—H42C	0.9600
C11—H11	0.9300	C44—C45	1.390 (3)
C12—C13	1.389 (4)	C44—C49	1.391 (3)
C12—H12	0.9300	C45—C46	1.385 (3)
C13—C14	1.387 (4)	C45—H45	0.9300
C13—C17	1.514 (3)	C46—C47	1.374 (4)
C14—C15	1.394 (3)	C46—H46	0.9300
C14—H14	0.9300	C47—C48	1.384 (4)
C15—C16	1.490 (3)	C47—C51	1.516 (3)
C16—H16A	0.9600	C48—C49	1.398 (3)
C16—H16B	0.9600	C48—H48	0.9300
C16—H16C	0.9600	C49—C50	1.492 (4)
C17—H17A	0.9600	C50—H50A	0.9600
C17—H17B	0.9600	C50—H50B	0.9600
C17—H17C	0.9600	C50—H50C	0.9600
C18—C19	1.478 (3)	C51—H51A	0.9600
C19—C20	1.320 (3)	C51—H51B	0.9600
C20—C21	1.456 (3)	C51—H51C	0.9600
C20—H20	0.9300		
			
C9—N1—C10	122.98 (19)	C25—C23—C21	120.5 (2)
C9—N1—H1A	115.2 (16)	C25—C23—C22	120.9 (2)
C10—N1—H1A	121.3 (16)	C21—C23—C22	60.85 (14)
C26—N2—C27	124.14 (18)	C25—C23—C24	113.8 (2)
C26—N2—H2A	116.9 (16)	C21—C23—C24	114.8 (2)
C27—N2—H2A	118.9 (16)	C22—C23—C24	116.1 (2)
C43—N3—C44	124.30 (19)	C23—C24—H24A	109.5
C43—N3—H3A	119.3 (16)	C23—C24—H24B	109.5
C44—N3—H3A	116.4 (16)	H24A—C24—H24B	109.5
F3'—C1—F1'	111.1 (7)	C23—C24—H24C	109.5
F3'—C1—F2	83.6 (6)	H24A—C24—H24C	109.5
F1'—C1—F2	123.1 (8)	H24B—C24—H24C	109.5
F3'—C1—F1	129.4 (7)	C23—C25—H25A	109.5
F1'—C1—F1	22.7 (7)	C23—C25—H25B	109.5
F2—C1—F1	107.4 (5)	H25A—C25—H25B	109.5
F3'—C1—F3	27.2 (6)	C23—C25—H25C	109.5
F1'—C1—F3	84.6 (6)	H25A—C25—H25C	109.5
F2—C1—F3	103.2 (5)	H25B—C25—H25C	109.5
F1—C1—F3	104.9 (5)	O2—C26—N2	122.58 (19)
F3'—C1—F2'	109.1 (8)	O2—C26—C22	123.05 (19)
F1'—C1—F2'	105.9 (8)	N2—C26—C22	114.37 (18)
F2—C1—F2'	26.4 (6)	C28—C27—C32	120.00 (19)
F1—C1—F2'	85.8 (7)	C28—C27—N2	120.8 (2)
F3—C1—F2'	124.4 (7)	C32—C27—N2	119.17 (19)
F3'—C1—C2	107.5 (7)	C29—C28—C27	120.9 (2)
F1'—C1—C2	112.6 (7)	C29—C28—H28	119.6
F2—C1—C2	114.1 (4)	C27—C28—H28	119.6
F1—C1—C2	111.6 (5)	C28—C29—C30	120.5 (2)
F3—C1—C2	114.8 (4)	C28—C29—H29	119.8
F2'—C1—C2	110.6 (6)	C30—C29—H29	119.8
C3—C2—C1	124.7 (3)	C29—C30—C31	118.3 (2)
C3—C2—Cl1'	114.3 (3)	C29—C30—C34	120.7 (3)
C1—C2—Cl1'	116.7 (2)	C31—C30—C34	121.0 (3)
C3—C2—Cl1	124.4 (2)	C30—C31—C32	123.1 (2)
C1—C2—Cl1	110.2 (2)	C30—C31—H31	118.5
Cl1'—C2—Cl1	32.3 (3)	C32—C31—H31	118.5
C2—C3—C4	125.0 (2)	C31—C32—C27	117.3 (2)
C2—C3—H3	117.5	C31—C32—C33	121.1 (2)
C4—C3—H3	117.5	C27—C32—C33	121.65 (19)
C3—C4—C6	122.1 (2)	C32—C33—H33A	109.5
C3—C4—C5	119.69 (19)	C32—C33—H33B	109.5
C6—C4—C5	59.57 (14)	H33A—C33—H33B	109.5
C3—C4—H4	114.8	C32—C33—H33C	109.5
C6—C4—H4	114.8	H33A—C33—H33C	109.5
C5—C4—H4	114.8	H33B—C33—H33C	109.5
C9—C5—C6	124.1 (2)	C30—C34—H34A	109.5
C9—C5—C4	120.18 (18)	C30—C34—H34B	109.5
C6—C5—C4	59.09 (15)	H34A—C34—H34B	109.5
C9—C5—H5	114.2	C30—C34—H34C	109.5
C6—C5—H5	114.2	H34A—C34—H34C	109.5
C4—C5—H5	114.2	H34B—C34—H34C	109.5
C7—C6—C4	119.8 (2)	F8—C35—F9	106.8 (2)
C7—C6—C8	114.0 (2)	F8—C35—F7	106.5 (2)
C4—C6—C8	116.0 (2)	F9—C35—F7	106.4 (2)
C7—C6—C5	120.94 (19)	F8—C35—C36	112.3 (2)
C4—C6—C5	61.34 (15)	F9—C35—C36	112.2 (2)
C8—C6—C5	114.9 (2)	F7—C35—C36	112.2 (2)
C6—C7—H7A	109.5	C37—C36—C35	124.3 (2)
C6—C7—H7B	109.5	C37—C36—Cl3	123.44 (19)
H7A—C7—H7B	109.5	C35—C36—Cl3	112.24 (17)
C6—C7—H7C	109.5	C36—C37—C38	125.0 (2)
H7A—C7—H7C	109.5	C36—C37—H37	117.5
H7B—C7—H7C	109.5	C38—C37—H37	117.5
C6—C8—H8A	109.5	C37—C38—C40	122.90 (19)
C6—C8—H8B	109.5	C37—C38—C39	121.05 (18)
H8A—C8—H8B	109.5	C40—C38—C39	59.66 (14)
C6—C8—H8C	109.5	C37—C38—H38	114.2
H8A—C8—H8C	109.5	C40—C38—H38	114.2
H8B—C8—H8C	109.5	C39—C38—H38	114.2
O1—C9—N1	122.2 (2)	C43—C39—C40	121.59 (18)
O1—C9—C5	124.4 (2)	C43—C39—C38	123.25 (19)
N1—C9—C5	113.4 (2)	C40—C39—C38	59.84 (14)
C11—C10—C15	120.8 (2)	C43—C39—H39	113.9
C11—C10—N1	119.1 (2)	C40—C39—H39	113.9
C15—C10—N1	120.1 (2)	C38—C39—H39	113.9
C12—C11—C10	120.3 (2)	C41—C40—C39	121.13 (18)
C12—C11—H11	119.8	C41—C40—C42	113.58 (19)
C10—C11—H11	119.8	C39—C40—C42	115.55 (19)
C11—C12—C13	120.6 (2)	C41—C40—C38	119.7 (2)
C11—C12—H12	119.7	C39—C40—C38	60.50 (14)
C13—C12—H12	119.7	C42—C40—C38	116.51 (18)
C14—C13—C12	118.0 (2)	C40—C41—H41A	109.5
C14—C13—C17	121.0 (2)	C40—C41—H41B	109.5
C12—C13—C17	121.0 (2)	H41A—C41—H41B	109.5
C13—C14—C15	123.0 (2)	C40—C41—H41C	109.5
C13—C14—H14	118.5	H41A—C41—H41C	109.5
C15—C14—H14	118.5	H41B—C41—H41C	109.5
C14—C15—C10	117.1 (2)	C40—C42—H42A	109.5
C14—C15—C16	120.9 (2)	C40—C42—H42B	109.5
C10—C15—C16	122.0 (2)	H42A—C42—H42B	109.5
C15—C16—H16A	109.5	C40—C42—H42C	109.5
C15—C16—H16B	109.5	H42A—C42—H42C	109.5
H16A—C16—H16B	109.5	H42B—C42—H42C	109.5
C15—C16—H16C	109.5	O3—C43—N3	123.50 (19)
H16A—C16—H16C	109.5	O3—C43—C39	123.32 (19)
H16B—C16—H16C	109.5	N3—C43—C39	113.15 (19)
C13—C17—H17A	109.5	C45—C44—C49	120.8 (2)
C13—C17—H17B	109.5	C45—C44—N3	119.6 (2)
H17A—C17—H17B	109.5	C49—C44—N3	119.5 (2)
C13—C17—H17C	109.5	C46—C45—C44	119.8 (2)
H17A—C17—H17C	109.5	C46—C45—H45	120.1
H17B—C17—H17C	109.5	C44—C45—H45	120.1
F6—C18—F5	107.1 (2)	C47—C46—C45	121.2 (3)
F6—C18—F4	106.2 (2)	C47—C46—H46	119.4
F5—C18—F4	105.6 (2)	C45—C46—H46	119.4
F6—C18—C19	112.3 (2)	C46—C47—C48	118.0 (2)
F5—C18—C19	112.8 (2)	C46—C47—C51	121.5 (3)
F4—C18—C19	112.3 (2)	C48—C47—C51	120.6 (3)
C20—C19—C18	123.9 (2)	C47—C48—C49	123.1 (2)
C20—C19—Cl2	123.72 (18)	C47—C48—H48	118.5
C18—C19—Cl2	112.34 (18)	C49—C48—H48	118.5
C19—C20—C21	125.4 (2)	C44—C49—C48	117.1 (2)
C19—C20—H20	117.3	C44—C49—C50	122.1 (2)
C21—C20—H20	117.3	C48—C49—C50	120.8 (2)
C20—C21—C23	121.1 (2)	C49—C50—H50A	109.5
C20—C21—C22	123.12 (17)	C49—C50—H50B	109.5
C23—C21—C22	59.62 (14)	H50A—C50—H50B	109.5
C20—C21—H21	114.1	C49—C50—H50C	109.5
C23—C21—H21	114.1	H50A—C50—H50C	109.5
C22—C21—H21	114.1	H50B—C50—H50C	109.5
C26—C22—C23	123.0 (2)	C47—C51—H51A	109.5
C26—C22—C21	120.64 (17)	C47—C51—H51B	109.5
C23—C22—C21	59.53 (14)	H51A—C51—H51B	109.5
C26—C22—H22	114.3	C47—C51—H51C	109.5
C23—C22—H22	114.3	H51A—C51—H51C	109.5
C21—C22—H22	114.3	H51B—C51—H51C	109.5
			
F3'—C1—C2—C3	−137.2 (10)	C20—C21—C23—C24	−140.0 (2)
F1'—C1—C2—C3	−14.5 (11)	C22—C21—C23—C24	107.3 (2)
F2—C1—C2—C3	132.1 (7)	C26—C22—C23—C25	1.2 (3)
F1—C1—C2—C3	10.0 (7)	C21—C22—C23—C25	110.1 (2)
F3—C1—C2—C3	−109.1 (8)	C26—C22—C23—C21	−108.8 (2)
F2'—C1—C2—C3	103.8 (11)	C26—C22—C23—C24	146.1 (2)
F3'—C1—C2—Cl1'	67.7 (11)	C21—C22—C23—C24	−105.1 (2)
F1'—C1—C2—Cl1'	−169.6 (11)	C27—N2—C26—O2	5.7 (4)
F2—C1—C2—Cl1'	−23.0 (8)	C27—N2—C26—C22	−174.6 (2)
F1—C1—C2—Cl1'	−145.1 (8)	C23—C22—C26—O2	44.6 (3)
F3—C1—C2—Cl1'	95.8 (9)	C21—C22—C26—O2	−26.9 (3)
F2'—C1—C2—Cl1'	−51.4 (12)	C23—C22—C26—N2	−135.1 (2)
F3'—C1—C2—Cl1	33.0 (10)	C21—C22—C26—N2	153.5 (2)
F1'—C1—C2—Cl1	155.8 (10)	C26—N2—C27—C28	46.4 (3)
F2—C1—C2—Cl1	−57.7 (7)	C26—N2—C27—C32	−136.8 (2)
F1—C1—C2—Cl1	−179.7 (6)	C32—C27—C28—C29	0.5 (4)
F3—C1—C2—Cl1	61.2 (8)	N2—C27—C28—C29	177.3 (2)
F2'—C1—C2—Cl1	−86.0 (11)	C27—C28—C29—C30	−0.2 (4)
C1—C2—C3—C4	−179.3 (2)	C28—C29—C30—C31	−0.7 (4)
Cl1'—C2—C3—C4	−23.7 (5)	C28—C29—C30—C34	179.7 (3)
Cl1—C2—C3—C4	11.8 (5)	C29—C30—C31—C32	1.3 (4)
C2—C3—C4—C6	−168.2 (3)	C34—C30—C31—C32	−179.1 (3)
C2—C3—C4—C5	121.1 (3)	C30—C31—C32—C27	−1.0 (4)
C3—C4—C5—C9	−2.0 (3)	C30—C31—C32—C33	179.3 (3)
C6—C4—C5—C9	−114.0 (2)	C28—C27—C32—C31	0.0 (3)
C3—C4—C5—C6	112.0 (3)	N2—C27—C32—C31	−176.8 (2)
C3—C4—C6—C7	3.2 (3)	C28—C27—C32—C33	179.8 (2)
C5—C4—C6—C7	111.2 (2)	N2—C27—C32—C33	3.0 (3)
C3—C4—C6—C8	146.5 (2)	F8—C35—C36—C37	−119.2 (2)
C5—C4—C6—C8	−105.5 (2)	F9—C35—C36—C37	1.1 (3)
C3—C4—C6—C5	−108.0 (2)	F7—C35—C36—C37	120.9 (2)
C9—C5—C6—C7	−1.9 (3)	F8—C35—C36—Cl3	60.5 (2)
C4—C5—C6—C7	−109.5 (2)	F9—C35—C36—Cl3	−179.19 (16)
C9—C5—C6—C4	107.6 (2)	F7—C35—C36—Cl3	−59.5 (2)
C9—C5—C6—C8	−145.1 (2)	C35—C36—C37—C38	−176.9 (2)
C4—C5—C6—C8	107.3 (2)	Cl3—C36—C37—C38	3.5 (3)
C10—N1—C9—O1	−5.1 (3)	C36—C37—C38—C40	−125.4 (2)
C10—N1—C9—C5	173.88 (19)	C36—C37—C38—C39	162.8 (2)
C6—C5—C9—O1	−37.7 (3)	C37—C38—C39—C43	2.4 (3)
C4—C5—C9—O1	33.3 (3)	C40—C38—C39—C43	−110.1 (2)
C6—C5—C9—N1	143.3 (2)	C37—C38—C39—C40	112.5 (2)
C4—C5—C9—N1	−145.6 (2)	C43—C39—C40—C41	3.9 (3)
C9—N1—C10—C11	111.4 (3)	C38—C39—C40—C41	−108.9 (2)
C9—N1—C10—C15	−71.4 (3)	C43—C39—C40—C42	−139.9 (2)
C15—C10—C11—C12	−1.2 (4)	C38—C39—C40—C42	107.3 (2)
N1—C10—C11—C12	176.0 (2)	C43—C39—C40—C38	112.8 (2)
C10—C11—C12—C13	−0.2 (4)	C37—C38—C40—C41	1.7 (3)
C11—C12—C13—C14	1.7 (4)	C39—C38—C40—C41	111.2 (2)
C11—C12—C13—C17	−177.0 (2)	C37—C38—C40—C39	−109.5 (2)
C12—C13—C14—C15	−1.8 (4)	C37—C38—C40—C42	144.8 (2)
C17—C13—C14—C15	176.9 (2)	C39—C38—C40—C42	−105.8 (2)
C13—C14—C15—C10	0.4 (4)	C44—N3—C43—O3	9.0 (4)
C13—C14—C15—C16	−179.8 (2)	C44—N3—C43—C39	−169.05 (19)
C11—C10—C15—C14	1.1 (4)	C40—C39—C43—O3	−43.1 (3)
N1—C10—C15—C14	−176.1 (2)	C38—C39—C43—O3	29.3 (3)
C11—C10—C15—C16	−178.7 (2)	C40—C39—C43—N3	134.9 (2)
N1—C10—C15—C16	4.1 (4)	C38—C39—C43—N3	−152.7 (2)
F6—C18—C19—C20	−2.9 (4)	C43—N3—C44—C45	45.3 (3)
F5—C18—C19—C20	118.3 (3)	C43—N3—C44—C49	−138.2 (2)
F4—C18—C19—C20	−122.5 (3)	C49—C44—C45—C46	0.4 (4)
F6—C18—C19—Cl2	178.17 (18)	N3—C44—C45—C46	176.9 (2)
F5—C18—C19—Cl2	−60.7 (3)	C44—C45—C46—C47	−1.4 (4)
F4—C18—C19—Cl2	58.5 (2)	C45—C46—C47—C48	1.6 (4)
C18—C19—C20—C21	−175.4 (2)	C45—C46—C47—C51	−178.3 (3)
Cl2—C19—C20—C21	3.4 (3)	C46—C47—C48—C49	−0.8 (4)
C19—C20—C21—C23	143.6 (2)	C51—C47—C48—C49	179.1 (3)
C19—C20—C21—C22	−144.6 (2)	C45—C44—C49—C48	0.4 (4)
C20—C21—C22—C26	3.2 (3)	N3—C44—C49—C48	−176.2 (2)
C23—C21—C22—C26	112.7 (2)	C45—C44—C49—C50	−178.5 (2)
C20—C21—C22—C23	−109.5 (3)	N3—C44—C49—C50	5.0 (4)
C20—C21—C23—C25	2.0 (3)	C47—C48—C49—C44	−0.2 (4)
C22—C21—C23—C25	−110.7 (2)	C47—C48—C49—C50	178.7 (3)
C20—C21—C23—C22	112.7 (2)		

### Hydrogen-bond geometry (Å, °)

**Table:**

*D*—H···*A*	*D*—H	H···*A*	*D*···*A*	*D*—H···*A*
N1—H1A···O2	0.90 (1)	1.95 (1)	2.840 (2)	171 (2)
N2—H2A···O3	0.91	1.99	2.893 (2)	174
N3—H3A···O1^i^	0.90	2.10	2.980 (2)	167

Symmetry codes: (i) *x*, *y*−1, *z*.
